# Parvalbumin-Expressing Neurons in the Nucleus Accumbens: A New Player in Amphetamine Sensitization and Reward

**DOI:** 10.1038/npp.2017.256

**Published:** 2017-11-22

**Authors:** Brandon L Warren, Leslie R Whitaker

**Affiliations:** 1 Behavioral Neuroscience Branch, IRP/NIDA/NIH/DHHS, Baltimore, MD, USA

Stimulants, including amphetamines, are widely abused in the United States and worldwide. Amphetamine acts on the dopamine transporter to elevate and prolong extracellular dopamine levels in the nucleus accumbens that mediate the drug’s rewarding effects. Repeated exposure to amphetamine has profound effects on the plasticity of medium spiny neurons (MSNs) in the nucleus accumbens that are thought to contribute to long-lasting behavioral alterations underlying amphetamine abuse. MSNs make up the vast majority of the neurons in the nucleus accumbens, but their activity is regulated by several types of interneurons, including fast-spiking parvalbumin expressing (PV+) interneurons. Although PV+ interneurons make up only 1–2% of neurons in the nucleus accumbens, they provide powerful feed-forward inhibitory control of MSN firing. Although their effects on nucleus accumbens electrophysiology are well understood, the role of accumbens PV+ interneurons in behavior has been far less explored.

In this issue of *Neuropsychopharmacology*, Wang *et al* (2017) tested the necessity of PV+ interneurons in amphetamine-induced locomotor sensitization and conditioned place preference (CPP). Locomotor sensitization refers to the observation that rodents become significantly more hyperactive in response to amphetamine after repeated exposures. CPP refers to a Pavlovian learning procedure where rodents will prefer to spend time in contexts in which they have received drugs. The authors used transgenic *Pvalb-*T2A-Cre mice to selectively silence PV+ interneurons with tetanus toxin light-chain (TeLC) in the nucleus accumbens. Tetanus toxin light-chain cleaves synaptobrevin, eliminating vesicle release and effectively turning off PV+ interneuron signaling. Silencing these interneurons profoundly disrupted both locomotor sensitization and CPP for amphetamine.

One goal of the study by Wang *et al* (2017) was to determine whether silencing PV+ neurons would influence locomotor sensitization to amphetamine. Importantly, by silencing PV+ neurons in the nucleus accumbens, the authors prevented locomotor sensitization to amphetamine, without altering the acute locomotor effects of the drug. A major strength of this study is that the authors tested the effect of inactivating PV+ neurons both during and after locomotor sensitization to amphetamine. In the first instance, the mice failed to develop locomotor sensitization during the ‘induction’ phase, as one might expect. However, inactivating PV+ interneurons in mice that had already developed locomotor sensitization returned these mice to un-sensitized levels of locomotion. This suggests that PV interneuron activity specifically affects the neural alterations required for amphetamine-induced locomotor sensitization without altering neural mechanisms underlying the drug’s acute effects on behavior.

Furthermore, Wang *et al* (2017) find that inhibition of PV neuron signaling in the nucleus accumbens prevents the development of CPP for amphetamine. CPP can be conceptualized as a measurement of the rewarding properties of a drug or alternatively as a measurement of the strength of association between a specific environmental context and reward. Given that the authors observe no difference in sucrose preference following PV neuron silencing, it is more likely that inhibition of PV neurons signaling impairs the development or expression of the learned association between the drug-associated context and drug reward, possibly by introducing noise into the system via disinhibition of a large population of nucleus accumbens MSNs.

Inhibition of PV+ interneuron signaling was also associated with a large increase in Fos expression in the nucleus accumbens, confirming that PV+ neurons in the nucleus accumbens regulate the activity of MSNs. Increased Fos expression in the nucleus accumbens has previously been associated with locomotor sensitization to amphetamine (Hedou *et al*, 2002), and Fos-expressing neurons, which include PV+ neurons, in the nucleus accumbens have been shown to form neuronal ensembles that mediate cocaine-induced locomotor sensitization (Koya *et al*, 2009). However, Wang *et al* (2017) are the first to examine the behavioral consequence of removing PV-mediated inhibition and enhancing MSN activity. Increasing the activity of all nucleus accumbens MSNs non-specifically likely introduces informational ‘noise’ into the system that reduces the specificity of the neuronal ensembles that are normally selectively activated to mediate amphetamine-induced locomotor sensitization and CPP. The authors show that impaired locomotor sensitization was not due to changes in amphetamine-induced dopamine release because silencing the activity of PV+ neurons in the nucleus accumbens does not affect amphetamine-induced dopamine release in this brain region. This suggests that PV+ interneurons have a role in enhancing the specificity of information processed by the nucleus accumbens by reducing the activity of less active neurons during behavior. Although PV+ interneurons make up only 1–2% of neurons in the nucleus accumbens, a previous study found that they represent >10% of Fos-expressing neurons associated with relapse to cocaine seeking (Cruz *et al*, 2014). The data presented in this issue hint that the reason these ensembles associated with cocaine seeking are enriched in PV+ neurons might be because PV+ neurons are critical for governing the specificity of MSN activity and enabling the mechanistic resolution required for highly specific drug-related learned associations to form (Warren *et al*, 2017). This is an unexplored line of research that might help explain how neuronal ensembles can encode highly specific learned associations.

The authors discuss the possibility of plasticity in nucleus accumbens microcircuitry as a cellular substrate for the expression of addiction-like behaviors. A previous study by Winters *et al* (2012) identified increased intrinsic excitability in CB1-receptor contained fast-spiking interneurons following withdrawal from repeated cocaine exposure. Given that a large proportion of these FSIs are PV+ neurons, it is possible that repeated amphetamine exposure may induce similar changes in intrinsic properties of these neurons. An important question for future exploration is how both intrinsic and synaptic plasticity in PV+ neurons in the nucleus accumbens contribute to the development and expression of addiction-like behaviors.

The results of Wang *et al* (2017) represent an important step in examining the role of PV+ neurons in drug-related behavior. However, many questions remain. For example, PV+ neurons appear to have a role in enhancing selectivity of neuronal ensembles; however, the mechanism underlying this function remains unknown. Furthermore, Wang *et al* (2017) convincingly demonstrates a role for PV+ interneurons in CPP, a procedure in which drug exposure is not under the control of the rat. Thus it is unknown whether these neurons would also mediate operant responding for amphetamine. Nevertheless, the current results provide critical evidence that PV+ interneurons have a significant role in drug-related learning.

## Funding and disclosure

BLW and LRW are supported by the National Institute on Drug Abuse Intramural Research Program. BLW was recently awarded a Pathway to Independence Award from the National Institute on Drug Abuse (1K99 DA042102). The authors declare no conflict of interest.
